# Health-Related Quality of Life and the Physical Activity Levels of Middle-Aged Women, California Health Interview Survey, 2005

**Published:** 2011-02-15

**Authors:** Cecily Luncheon, Matthew Zack

**Affiliations:** Centers for Disease Control and Prevention, Atlanta, Georgia; Health-Related Quality of Life Surveillance Team, Arthritis, Epilepsy and Quality of Life Branch, Division of Adult and Community Health, National Center for Chronic Disease Prevention and Health Promotion, Centers for Disease Control and Prevention

## Abstract

**Introduction:**

Several studies suggest that physical activity may improve health-related quality of life. Other studies have shown that participation in physical activity differs among women of different racial/ethnic groups. This study aimed to determine whether the association between physical activity and health-related quality of life differs among women aged 40 to 64 years from different racial/ethnic groups.

**Methods:**

We explored the association between physical activity level and health-related quality of life with descriptive statistics and multiple regression analyses adjusting for potential confounders among 11,887 women aged 40 to 64 years who identified themselves as Latinas, Asians, African Americans, or whites in the 2005 California Health Interview Survey.

**Results:**

Although white women reported more regular physical activity than women of other racial/ethnic groups, Asian women reported fewer mentally and overall unhealthy days than women of other groups. Nonetheless, as physical activity increased, health-related quality of life improved only among white women (fewer physically unhealthy, mentally unhealthy, recent activity limitation, and overall unhealthy days) and among Latinas (fewer overall unhealthy days).

**Conclusion:**

Future studies should try to confirm if and clarify why the association between physical activity level and health-related quality of life differs among these middle-aged women of different races/ethnicities. If confirmed, this association would imply that health care professionals and those who design public health interventions may need to vary their promotion methods and messages to encourage physical activity among women of different races/ethnicities.

## Introduction

Physical activity improves overall health and helps to prevent and reduce stress and many chronic diseases including cardiovascular diseases, type 2 diabetes, arthritis, and some forms of cancer (eg, breast and colon) (1). Getting people to be physically active is a major public health concern (2). As women age, their levels of physical activity decrease (3,4), and more than 60% of women older than 65 years do not participate in recommended levels of physical activity (5). Although general national health education plans exist for all adults aged 18 to 64 years, no national health education campaign specifically targets women in midlife to engage in physical activity before they enter their senior years.

Levels of participation in physical activity differ by race/ethnicity (6-10), age (3-5,11), and sex (12,13). Minority women participate less often in physical activity than do their white peers (14). Minority women are also at higher risk for several types of chronic diseases associated with being sedentary (eg, heart disease, type 2 diabetes, obesity) (15-17).

Physical activity may also improve health-related quality of life (HRQOL). For example, in one study of people with arthritis, those who were physically active reported fewer unhealthy days than those who were inactive (18). Physical activity also positively influences women with breast cancer after diagnosis, thus improving their HRQOL (19). Older women who were physically active and lived independently also reported better quality of life (5). In the US general adult population, those who were inactive were more likely to report experiencing poorer health than those who participated in the recommended amount of physical activity (20).

However, being physically active may have different effects on HRQOL for women of different racial/ethnic groups. Some women may be physically active because it helps prevent future disease and disability, while others may be active because it improves their immediate HRQOL. The association between physical activity among middle-aged women and self-reported HRQOL among ethnic groups in the United States has not been investigated in detail. We attempted to determine whether this association differs across 4 racial/ethnic groups after adjustment for potential confounders. If women in different racial/ethnic groups experience different degrees of HRQOL from the same amount of physical activity, this may explain why women in different racial/ethnic groups vary in their participation in leisure-time physical activity. This finding may also support new strategies for health promotion and intervention targeted to different racial/ethnic groups. The purpose of this research is to determine whether the association between physical activity and HRQOL differs among California's Latina, Asian, African American, and white women aged 40 to 64 years.

## Methods

We used secondary data from a cross-sectional study, the 2005 California Health Interview Survey (CHIS), a random-digit–dialed telephone survey in 5 languages completed every 2 years to observe the overall health status and other health-related characteristics of California residents. The University of California, Los Angeles, Center for Health Policy Research (UCLA-CHPR) administers CHIS. CHIS is suitable for this study's purpose because it has more than 600 women aged 40 to 64 years in each of the 4 racial/ethnic groups of interest and includes measures of physical activity and HRQOL and information about several sociodemographic and individual characteristics associated with both physical activity and HRQOL. The CHIS sample arises from multistage sampling based on 44 primary geographic sampling units identified in the first stage. Within each of these units, CHIS randomly selects and dials household telephone numbers and, at each number, chooses 1 adult (aged 18 y or older). In the 2005 CHIS, the response rate was 29.5%; 43,020 adult participants completed the survey (21).

### Sample

In this study, 12,408 women identified themselves as aged 40 to 64 years and answered questions about race and ethnicity. This study focused on the women who identified themselves as Latinas, Asians, African Americans, and whites. This study excluded from the analysis women from smaller racial/ethnic groups including American Indians or Alaska Natives (n = 184), Pacific Islanders (n = 31), and other single or multiple races (n = 306). The final sample included 11,887 participants (22).

### Physical activity

Women in CHIS responded to 4 questions about their levels of physical activity based on definitions of validated measures of vigorous and moderate activity used in the *Healthy People 2010* objectives (2). From these 4 questions, UCLA-CHPR created a composite variable that classified physical activity into 3 levels. Regular physical activity is either vigorous activity that makes one breathe much harder than normal for 20 or more minutes 3 or more days per week or moderate activity that makes one breathe somewhat harder than normal for 30 or more minutes 5 or more days per week. Some physical activity is either any vigorous activity less often than that for regular physical activity, any moderate activity at least 10 minutes but less often than that for regular physical activity, or walking at least 10 minutes for transportation or fun in the past week. No physical activity (being sedentary) is any activity not meeting the criteria for regular or some physical activity (22).

### Health-related quality of life

Women in CHIS also answered validated questions about their recent HRQOL relating to the past 30 days before their interview, which are also used in the Behavioral Risk Factor Surveillance System (23): physically unhealthy days ("On how many days was your physical health not good?"), mentally unhealthy days ("Mental health includes stress, feeling sad or not feeling like yourself. On how many days was your mental health not good?"), and recent activity limitation days ("On how many days did poor health keep you from doing your usual things, such as taking care of yourself, working, and having fun?"). We calculated a fourth measure, overall unhealthy days, as the sum of physically unhealthy days and mentally unhealthy days, with a logical maximum of 30 days.

### Covariates

For this study, other potential confounders associated with physical activity and these HRQOL measures adjusted for in the analysis included marital status, place of birth (in the United States or outside the United States), urban-rural place of residence, educational level, employment status, health insurance status, annual household income, self-rated health, and body mass index (BMI) (23-25).

### Data analysis

To account for complex survey and sample design, we analyzed the participants' responses by using SAS-callable SUDAAN version 9.1.3 (Research Triangle Institute, Research Triangle Park, North Carolina). To describe the characteristics of the sample, we calculated percentages and means weighted by the respondents' individual sampling weight that adjusted for nonresponse. We used multiple linear regression to identify significant predictors of HRQOL while controlling for covariates listed previously. We incorporated in these regressions an interaction term combining the racial/ethnic group and level of physical activity to determine whether the effect of physical activity level on HRQOL days differed by racial/ethnic group. We employed 4 models, one for each dependent HRQOL variable selected for this study. The primary outcomes are the average predicted values of these HRQOL dependent variables at each level of the independent variables and adjusted for the effects of all the other independent variables in the model (the predicted marginals and their 95% confidence intervals [CIs] [26]). We considered subgroups whose 95% CIs of these predicted marginals did not overlap to be significantly different from one another at *P* < .05.

## Results

### Sample characteristics

Based on weighted percentages, 60% of women in the sample were white, 20% were Latina, 14% were Asian, and 7% were African American (Table 1). Thirty-two percent of white women participated in regular physical activity, slightly more than the other 3 groups. Yet more Asian women had a normal BMI and fewer were obese compared with women in the other groups. White women rated their health as excellent more often than women in the other groups.

### Physically unhealthy days

Asian women reported fewer mean physically unhealthy days than Latina or African American women (Table 2). After adjustment for other covariates, more physically unhealthy days were associated with being unemployed, having health insurance, and reporting worse self-rated health (Table 3). Average physically unhealthy days increased with decreasing physical activity only for white women (5.2 days for being regularly active vs 8.1 days for being sedentary).

### Mentally unhealthy days

Asian women also reported fewer mentally unhealthy days than did women in the other racial/ethnic groups. More mentally unhealthy days were associated with having been previously married, being native-born, having a lower annual household income, and reporting worse self-rated health. Average mentally unhealthy days increased with decreasing physical activity mainly for white women (5.0 days for being regularly active vs 7.6 days for being sedentary).

### Recent activity limitation days

Asian women also reported fewer recent activity limitation days than white women and African American women but not Latinas. Being native-born, having more education, being unemployed, having a lower annual household income, and reporting worse self-rated health were related to more recent activity limitation days. Average recent activity limitation days increased with decreasing physical activity only for white women (2.5 days for being regularly active vs 5.3 days for being sedentary).

### Overall unhealthy days

Finally, Asian women reported the fewest overall unhealthy days. Not being currently married, being native-born, being unemployed, having a lower annual household income, and reporting worse self-rated health were associated with more average overall unhealthy days. Average overall unhealthy days increased with decreasing physical activity for Latinas (6.1 days for being regularly active vs 10.7 days for being sedentary) and white women (8.6 days for being regularly active vs 12.2 days for being sedentary) but not for Asian or African American women.

## Discussion

This study examined differences in the association between physical activity and HRQOL in women aged 40 to 64 years of different racial/ethnic groups. The findings that white women participated more often, and African American women less often, in regular physical activity agree with those of other studies (14). Because close to two-thirds of the African American women were not currently married, lack of available childcare for women with children, one of the main barriers to being more physically active (10,11,13,27), may account for the smaller percentage of African Americans participating in regular physical activity and the larger percentage of them being sedentary. However, lack of childcare would not explain the lack of physical activity among these older African American women, whose children may no longer live at home or may care for themselves, as well as among Latinas, who were more often currently married (28).

Lower educational levels and annual household incomes, another factor associated with less physical activity (10,29), did not affect physical activity levels among the women in this study because Asians and Latinas had similar levels of regular physical activity (26% vs 23%, respectively) despite having different educational levels (87% completing high school vs 50%, respectively) and annual household incomes (11% less than the federal poverty level vs 28%, respectively).

Asians reported fewer mentally unhealthy days and overall unhealthy days than did the other racial/ethnic groups. African Americans and Latinas reported more overall unhealthy days than did whites or Asians, and African Americans reported more recent activity limitation days than did the other groups. Yet only whites and Latinas who engaged in either regular or some physical activity reported better HRQOL than those who were sedentary. For whites, better HRQOL reflected all measures of unhealthy days, while for Latinas increasing physical activity was associated with better HRQOL only for overall unhealthy days. These findings are robust despite adjustment for self-rated health, a strong correlate for unhealthy days.

These study findings suggest that physical activity does not have the same effect on the HRQOL of middle-aged women in different racial/ethnic groups. Being physically active may not have the same meaning for each racial/ethnic group (8). Some groups may not consciously think of physical activity as part of their daily routine or may not consider it a separate act performed to achieve optimal health (30). Moreover, walking or riding a bike to work and working in factories or in the fields as many immigrants do may be perceived as a necessity, not a way to improve their mental and physical health. Even if such unintended and incidental physical activity improves mental and physical health physiologically, HRQOL, construed as perceived mental and physical health, may not improve. Finally, because women in different racial/ethnic groups may see different health care professionals, perhaps these professionals differ in their promotion of physical activity as important for health and HRQOL.

The study was subject to limitations. The response rate for the 2005 CHIS was only 30%, implying that this study's findings may not represent those of California adult women (21). For example, those who were more physically active may have been outside of their residence, engaging in physical activity, when CHIS attempted to call their home. Those with the poorest HRQOL may also have been less likely to participate in the survey. In addition, CHIS selects only landline telephone numbers for interview so that women who use only mobile telephones and who may have different physical activity habits would not have been sampled (21). Because more than two-thirds of the Latinas and Asian women in the survey were foreign-born, and because these women came from several countries with different languages, cultures, diet, and religions, the findings summarized for Latina and Asian groups may not reflect these country-specific cultural differences in how physical activity and HRQOL are perceived. Because CHIS is cross-sectional, determining whether physical activity affected HRQOL or HRQOL affected physical activity was not possible.

Despite these limitations, if physical activity levels truly affect HRQOL differently for women among different racial/ethnic groups, then programs that promote physical activity to women in different racial/ethnic groups may have to emphasize benefits to both health and HRQOL for some groups and benefits only to health for other groups. Even without considering HRQOL, health care professionals should discuss the concept of being physically active with all women. Further research is necessary to confirm this study's findings and to determine whether the concept of HRQOL is meaningful and relevant for all racial/ethnic groups. Finally, surveys may need to consider an expanded definition of physical activity beyond leisure-time activity to accommodate the physical activity involved in working.

## Figures and Tables

**Table 1:** Characteristics of Women Aged 40-64 Years (N = 11,887), by Race/Ethnicity, California Health Interview Survey, 2005

Characteristic	Race/Ethnicity, n (%)^a^			

	Latina, n = 1,441	Asian, n = 1,120	African American, n = 614	White, n = 8,712
**Marital status**				
Married	820 (64)	844 (79)	185 (36)	4,793 (63)
Widowed, separated, divorced, or living with partner	494 (28)	219 (17)	285 (42)	3,090 (29)
Never married	127 (8)	57 (4)	144 (22)	829 (8)
**Born outside United States**	902 (70)	954 (88)	41 ( 7)	729 (10)
**Place of residence**				
Large city	617 (53)	603 (58)	330 (62)	1,940 (31)
Small city	422 (24)	199 (16)	158 (19)	2,737 (28)
Suburban area	220 (15)	275 (23)	100 (15)	2,053 (24)
Town or rural area	182 (9)	43 (3)	26 (4)	1,982 (17)
**Education**				
Less than high school graduate	585 (50)	110 (13)	34 (8)	267 (4)
High school graduate, no college	325 (23)	214 (22)	141 (25)	1,648 (24)
Some college	359 (18)	207 (17)	242 (39)	2,785 (31)
College graduate	172 (9)	589 (49)	197 (28)	4,012 (41)
**Employed**	948 (63)	771 (70)	411 (66)	6,193 (71)
**Has health insurance**	1,096 (73)	931 (82)	556 (89)	8,029 (93)
**% Federal poverty level**				
0-99	365 (28)	130 (11)	85 (16)	412 (4)
100-199	411 (31)	171 (19)	103 (17)	838 (9)
200-299	181 (13)	126 (11)	76 (11)	881 (10)
≥300	484 (29)	693 (60)	350 (57)	6,581 (76)
**Physical activity level**				
Regular physical activity	359 (23)	293 (26)	143 (22)	2,915 (32)
Some physical activity	903 (66)	710 (65)	380 (61)	4,880 (56)
Sedentary	179 (11)	117 (9)	91 (17)	917 (12)
**Body mass index, kg/m^2^**				
<18.5 (Underweight)	10 (<1)	52 (4)	7 (<1)	197 (3)
18.5-24.9 (Normal weight)	430 (28)	802 (71)	164 (29)	4,283 (49)
25.0-29.9 (Overweight)	479 (34)	99 (19)	196 (30)	2,390 (27)
≥30.0 (Obese)	522 (38)	67 (7)	247 (41)	184 (21)
**Self-rated general health**				
Excellent	153 (10)	174 (15)	78 (13)	2,445 (27)
Very good	289 (16)	316 (30)	107 (28)	3,062 (35)
Good	449 (31)	344 (30)	197 (30)	1,946 (23)
Fair	409 (33)	213 (17)	128 (21)	878 (10)
Poor	141 (10)	73 (7)	41 (8)	381 (4)

a Percentages are based on weighted analysis to account for complex sample survey design. Respondents were assigned to racial/ethnic groups on the basis of their responses on the questionnaire and the classification method developed by the University of California, Los Angeles, Center for Health Policy Research (21).

**Table 2:** Health-Related Quality of Life Indicators for Women Aged 40-64 Years (N = 11,887), by Race/Ethnicity, California Health Interview Survey, 2005

**Race/Ethnicity**	Mean Physically Unhealthy Days^a^ (95% CI)	Mean Mentally Unhealthy Days^a^ (95% CI)	Mean Recent Activity Limitation Days^a^ (95% CI)	Mean Overall Unhealthy Days^a^ (95% CI)
Latina	6.1 (5.4-6.8)	5.7 (5.0-6.4)	2.3 (1.8-2.8)	9.9 (9.1-10.8)
Asian	3.9 (3.1-4.6)	3.8 (3.1-4.5)	1.5 (1.0-1.9)	6.7 (5.7-7.6)
African American	6.0 (4.9-7.1)	6.0 (5.0-7.0)	3.8 (3.0-4.2)	10.3 (9.0-11.6)
White	4.8 (4.5-5.2)	5.2 (4.9-5.5)	2.8 (2.6-3.0)	8.6 (8.3-9.0)

Abbreviation: CI, confidence interval.

a Means and 95% CIs are based on weighted analysis to account for complex sample survey design. Days are out of the previous 30 days. Respondents were assigned to racial/ethnic groups on the basis of their responses on the questionnaire and the classification method developed by the University of California, Los Angeles, Center for Health Policy Research (21).

**Table 3:** Health-Related Quality of Life Indicators for Women Aged 40-64 Years (N = 11,887), California Health Interview Survey, 2005

Characteristic	Mean Physically Unhealthy Days, PM (95% CI)^a^	Mean Mentally Unhealthy Days, PM (95% CI)^a^	Mean Recent Activity Limitation Days, PM (95% CI)^a^	Mean Overall Unhealthy Days, PM (95% CI)^a^
**Race/ethnicity**				
Latina	4.1 (3.5-4.6)	4.5 (3.9-5.1)	1.6 (1.2-2.0)	7.5 (6.9-8.2)
Asian	3.7 (3.0-4.5)	4.5 (3.8-5.2)	1.8 (1.3-2.2)	7.1 (6.3-7.9)
African American	4.7 (3.9-5.5)	4.8 (3.9-5.7)	2.9 (2.2-3.6)	8.3 (7.3-9.4)
White	5.7 (5.3-6.0)	5.6 (5.3-5.9)	3.1 (2.9-3.3)	9.5 (9.1-9.9)
**Marital status**				
Married	4.9 (4.6-5.1)	4.8 (4.5-5.1)	2.4 (2.2-2.6)	8.3 (8.0-8.7)
Widowed, separated, divorced, or living with partner	5.4 (5.0-5.7)	5.9 (5.5-6.3)	3.0 (2.6-3.3)	9.4 (8.9-9.8)
Never married	5.2 (4.5-5.8)	5.5 (4.8-6.2)	2.9 (2.5-3.3)	9.2 (8.4-10.0)
**Place of birth**				
United States	5.1 (4.8-5.4)	5.6 (5.3-5.9)	2.8 (2.6-3.0)	9.1 (8.7-9.5)
Outside United States	4.9 (4.5-5.3)	4.2 (3.8-4.7)	2.1 (1.8-2.5)	7.9 (7.4-8.4)
**Place of residence**				
Large city	5.1 (4.7-5.4)	5.2 (4.8-5.5)	2.6 (2.3-2.8)	8.9 (8.4-9.3)
Small city	4.9 (4.4-5.3)	5.2 (4.8-5.6)	2.7 (2.3-3.0)	8.5 (8.0-8.9)
Suburban area	5.1 (4.7-5.6)	5.1 (4.6-5.6)	2.4 (2.2-2.7)	8.8 (8.2-9.4)
Town or rural area	5.0 (4.5-5.5)	5.2 (4.7-5.7)	2.8 (2.4-3.2)	8.4 (7.8-9.1)
**Education**				
Less than high school graduate	4.5 (3.6-5.3)	5.1 (4.2-6.1)	1.9 (1.3-2.5)	8.1 (7.2-9.1)
High school graduate, no college	4.7 (4.3-5.0)	5.3 (4.8-5.8)	2.3 (2.0-2.6)	8.6 (8.0-9.1)
Some college	5.4 (5.0-5.8)	5.4 (5.0-5.8)	2.9 (2.6-3.2)	9.3 (8.8-9.8)
College graduate	5.2 (4.8-5.5)	4.9 (4.6-5.2)	2.9 (2.6-3.1)	8.6 (8.2-9.0)
**Employment**				
Employed	4.5 (4.3-4.8)	5.1 (4.8-5.4)	2.0 (1.8-2.2)	8.3 (8.0-8.7)
Unemployed	6.1 (5.7-6.5)	5.3 (4.9-5.8)	3.9 (3.6-4.2)	9.5 (9.0-10.1)
**Has health insurance**				
Yes	5.2 (4.9-5.4)	5.1 (4.9-5.4)	2.7 (2.5-2.8)	8.7 (8.4-9.1)
No	4.1 (3.5-4.8)	5.5 (4.8-6.1)	2.2 (1.7-2.7)	8.5 (7.7-9.3)
**% Federal poverty level**				
0-99	6.0 (5.0-7.1)	6.7 (5.6-7.7)	3.6 (3.0-4.1)	10.0 (8.9-11.2)
100-199	5.1 (4.5-5.8)	5.3 (4.7-5.9)	2.7 (2.2-3.2)	9.1 (8.3-9.8)
200-299	4.9 (4.3-5.5)	5.5 (4.8-6.1)	2.4 (1.9-2.8)	8.9 (8.1-9.8)
≥300	4.8 (4.6-5.1)	4.8 (4.5-5.1)	2.5 (2.2-2.7)	8.4 (8.0-8.7)
**Physical activity level**				
Regular physical activity	4.5 (4.2-4.9)	4.8 (4.4-5.2)	2.3 (2.0-2.5)	7.9 (7.5-8.4)
Some physical activity	4.9 (4.6-5.2)	5.0 (4.7-5.3)	2.5 (2.3-2.7)	8.6 (8.3-8.9)
Sedentary	6.9 (6.1-7.6)	6.9 (6.1-7.7)	4.0 (3.5-4.6)	11.1 (10.2-11.9)
**Self-rated health**				
Excellent	1.5 (1.1-1.8)	2.9 (2.5-3.2)	0.8 (0.6-1.0)	4.1 (3.7-4.5)
Very good	2.5 (2.2-2.7)	3.9 (3.6-4.2)	1.3 (1.1-1.5)	6.0 (5.7-6.4)
Good	4.6 (4.1-5.0)	5.2 (4.7-5.6)	2.3 (2.1-2.6)	8.8 (8.3-9.4)
Fair	10.0 (9.2-10.7)	7.8 (7.1-8.5)	5.0 (4.4-5.5)	14.5 (13.7-15.4)
Poor	19.1 (17.7-20.5)	12.5 (11.0-14.0)	10.5 (9.3-11.6)	22.4 (21.1-23.7)
**Body mass index, kg/m^2^**				
<18.5 (Underweight)	4.7 (3.8-5.6)	5.5 (4.4-6.7)	2.4 (1.5-3.3)	8.3 (7.1-9.6)
18.5-24.9 (Normal weight)	4.9 (4.6-5.2)	5.0 (4.7-5.3)	2.6 (2.4-2.8)	8.5 (8.1-8.9)
25.0-29.9 (Overweight)	5.0 (4.6-5.4)	5.0 (4.6-5.4)	2.6 (2.3-2.9)	8.6 (8.1-9.1)
≥30.0 (Obese)	5.3 (4.8-5.8)	5.6 (5.1-6.0)	2.6 (2.2-3.0)	9.3 (8.8-9.9)
** Physical activity level by race**				
**Latina**				
Regular physical activity	3.5 (2.6-4.3)	3.6 (2.5-4.6)	1.7 (1.0-2.4)	6.1 (5.0-7.2)
Some physical activity	4.1 (3.3-4.9)	4.5 (3.7-5.2)	1.5 (1.0-1.9)	7.6 (6.8-8.4)
Sedentary	5.2 (3.3-7.2)	7.1 (4.6-9.5)	2.0 (0.8-3.1)	10.7 (8.2-13.1)
**Asian**				
Regular physical activity	3.5 (2.7-4.3)	4.5 (3.6-5.5)	1.7 (1.2-2.3)	6.8 (5.6-7.9)
Some physical activity	3.7 (2.7-4.8)	4.7 (3.9-5.5)	1.8 (1.2-2.4)	7.2 (6.2-8.2)
Sedentary	4.4 (2.8-5.9)	4.0 (2.5-5.4)	1.6 (0.5-2.7)	7.3 (5.4-9.2)
**African American**				
Regular physical activity	3.8 (2.9-4.6)	6.9 (5.0-8.8)	3.0 (1.6-4.4)	9.2 (7.2-11.2)
Some physical activity	4.9 (3.7-6.1)	3.5 (2.5-4.5)	2.5 (1.6-3.4)	7.6 (6.4-8.9)
Sedentary	5.5 (3.5-7.6)	6.3 (3.7-8.9)	4.3 (1.7-6.8)	9.7 (7.5-11.9)
**White**				
Regular physical activity	5.2 (4.7-5.6)	5.0 (4.6-5.4)	2.5 (2.2-2.7)	8.6 (8.1-9.2)
Some physical activity	5.4 (5.1-5.7)	5.5 (5.1-5.8)	3.0 (2.7-3.2)	9.4 (9.0-9.8)
Sedentary	8.1 (7.2-9.0)	7.6 (6.6-8.6)	5.3 (4.4-6.1)	12.2 (11.2-13.2)

Abbreviations: PM, predicted marginal; CI, confidence interval.

a PMs are the average values of the health-related quality of life measures in a subgroup adjusted for other variables in the model, and their 95% CIs are based on a weighted analysis to account for complex sample survey design. Days are out of the previous 30 days. Respondents were assigned to racial/ethnic groups on the basis of their responses on the questionnaire and the classification method developed by the University of California, Los Angeles, Center for Health Policy Research (21).
